# Bone metastases with “false negative” findings on ^18^F-FDG PET/CT in patients with angiosarcoma: A case series with literature review

**DOI:** 10.1097/MD.0000000000034196

**Published:** 2023-07-14

**Authors:** Akane Ariga, Seiichi Matsumoto, Taisuke Tanizawa, Keiko Hayakawa, Yusuke Minami, Masanori Saito, Norio Kurosawa, Kyoko Yamashita, Takashi Terauchi, Keisuke Ae

**Affiliations:** a Department of Orthopedic Oncology, Cancer Institute Hospital of the Japanese Foundation for Cancer Research, Tokyo, Japan; b Department of Pathology, Cancer Institute Hospital of the Japanese Foundation for Cancer Research, Tokyo, Japan; c Department of Nuclear Medicine, Cancer Institute Hospital of the Japanese Foundation for Cancer Research, Tokyo, Japan.

**Keywords:** ^18^F-FDG PET/CT, angiosarcoma, bone metastasis

## Abstract

Fluorine-18 fluorodeoxyglucose positron emission tomography/computed tomography (^18^F-FDG PET/CT) is considered a reliable and indispensable imaging method when evaluating distant metastases and clinical staging of angiosarcomas. Here, we report 2 cases of angiosarcoma with bone metastases with “false negative” findings on ^18^F-FDG PET/CT.

**Patient concerns:**

Case 1, a 39-year-old woman, who had undergone mastectomy for primary angiosarcoma 2 years prior, presented with a 5-month history of right coxalgia. Case 2 was a 37-year-old woman, who had undergone mastectomy for primary angiosarcoma 4 months prior. During postoperative follow-up, multiple bone lesions were detected on magnetic resonance imaging (MRI).

**Diagnoses:**

Based on the histopathological findings, both cases were diagnosed with bone metastases of angiosarcoma. Although MRI showed multiple bone metastatic lesions, ^18^F-FDG PET/CT showed no uptake or osteolytic destruction in both cases.

**Interventions:**

Weekly paclitaxel was initiated as a salvage chemotherapy in both cases.

**Outcomes:**

No uptake or osteolytic lesions were observed on ^18^F-FDG PET/CT, despite multiple bone metastases detected on MRI.

**Lessons:**

False-negative findings on ^18^F-FDG PET/CT should be considered when evaluating bone metastases of angiosarcoma. Even with negative findings on ^18^F-FDG PET/CT, open biopsy should be performed if MRI indicates bone metastases.

## 1. Introduction

Angiosarcoma is a malignant soft tissue tumor of vascular origin that accounts for 1% to 2% of all the soft tissue sarcomas.^[1,2]^ Angiosarcomas have poor prognosis with rapid progression and 5-year survival rate of approximately 10% to 50%.^[3,4]^ Surgery, tumor localization, tumor size, performance status, and histopathology have been reported as prognostic factors for angiosarcoma, among which the presence of distant metastasis at the initial diagnosis is reported to be an independent prognostic factor for the overall survival rate.^[5,6]^ Reportedly, angiosarcomas can easily metastasize to other parts of the body through the blood or lymphatic stream,^[7]^ especially in metastatic angiosarcomas, wherein 42.6% of patients developed bone metastases.^[8]^

Although fluorine-18 fluorodeoxyglucose positron emission tomography/computed tomography (^18^F-FDG PET/CT) is regarded as a reliable and indispensable imaging method for evaluating and staging bone metastases of angiosarcoma,^[9–14]^ here we report 2 cases of angiosarcoma with bone metastases that presented with “false negative” findings on ^18^F-FDG PET/CT. To the best of our knowledge, there has been only 1 report of false-negative findings of bone metastases of angiosarcoma on ^18^F-FDG PET/CT.

## 2. Case presentation

### 2.1. Case 1

**Figure 1.**
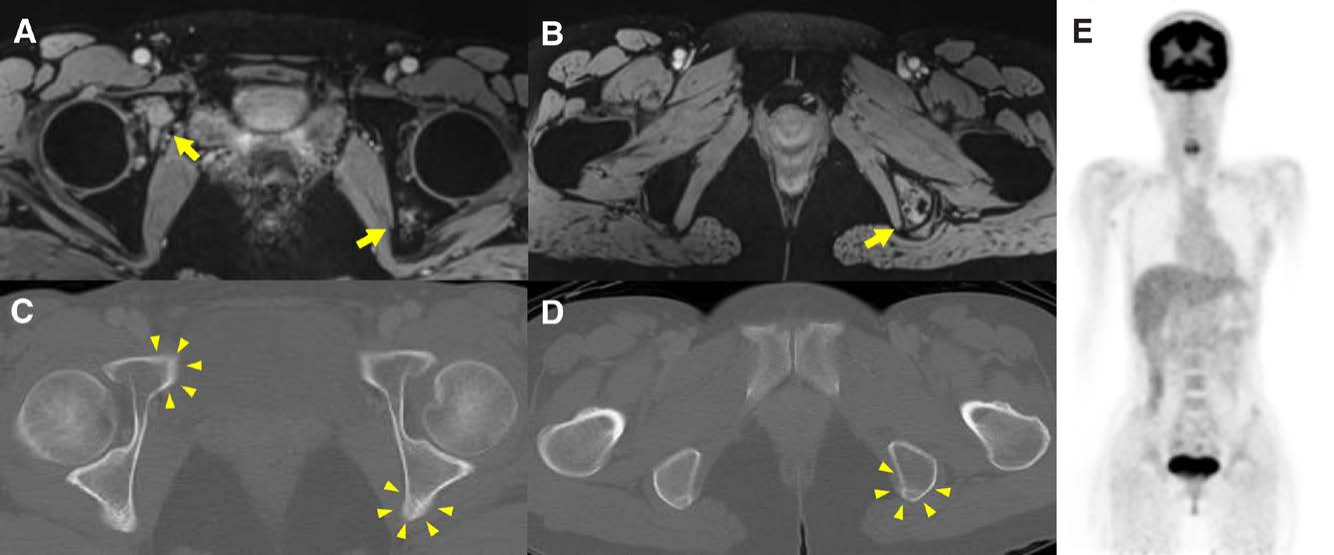
A 39-year-old woman was diagnosed with angiosarcoma of the breast and underwent mastectomy. One year after the surgery, she noticed worsening right coxalgia. On axial Gd-enhanced magnetic resonance imaging (MRI), multiple bone lesions in bilateral acetabulum (A) and the left ischium (B) (yellow arrows) strongly suggested bone metastases. However, axial fluorine-18 fluorodeoxyglucose positron emission tomography/computed tomography (^18^F-FDG PET/CT) showed neither osteolytic destruction nor abnormal uptake around the bilateral acetabulum (C) and the left ischium (D) (yellow arrowheads). Maximum intensity projection also suggested no findings of bone metastases (E).

**Figure 2.**
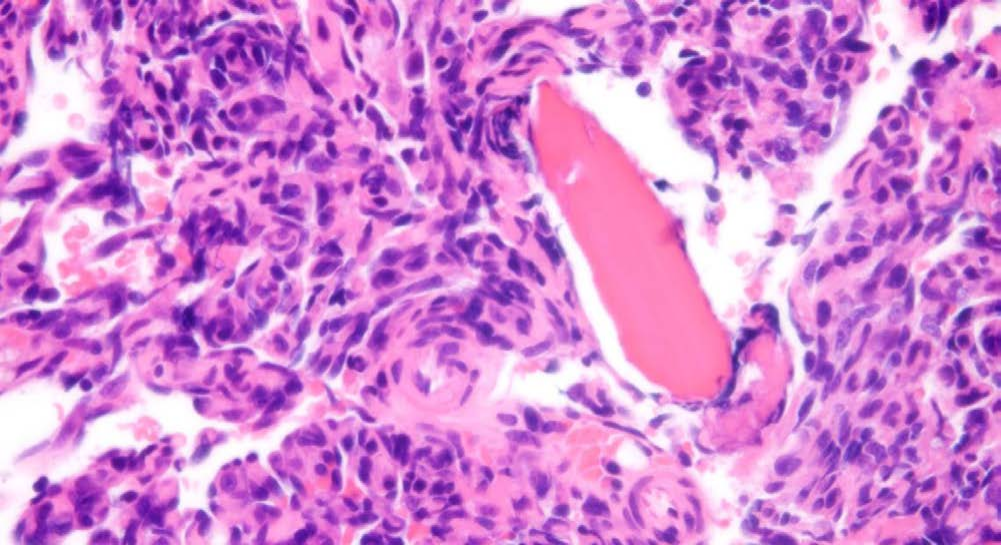
Hematoxylin and eosin stained specimen (×400) collected by open biopsy of the left ischiatic lesion revealed irregularly formed vascular channels covered by atypical endothelial cells, which is consistent with the metastasis of primary angiosarcoma of the breast.

A 39-year-old woman, who had no history of diabetes mellitus, presented with right coxalgia that had persisted for approximately 5 months. The patient first noticed a mass in her right breast 2 years prior, which had subsequently enlarged. One year later, the patient was diagnosed with angiosarcoma of the breast and underwent a mastectomy. Another year later, right coxalgia gradually appeared. Since there were no abnormal findings on radiographs, magnetic resonance imaging (MRI) was performed. Although MRI indicated bone lesions in the right acetabulum and left ischium (Fig. 1A and B), bone scintigraphy and ^18^F-FDG PET/CT revealed no abnormal uptake or osteolytic destruction (Fig. 1C–E). Therefore, the bone lesions were considered benign and were followed-up conservatively. Nevertheless, the patient’s right coxalgia worsened, and the patient was finally referred to our hospital. The multiple bone lesions detected on MRI were inconsistent with benign bone tumors and suggested the presence of bone metastases. There were no metastases present in other organs. An open biopsy of the left ischiatic lesion was conducted the following month. The pathological diagnosis was angiosarcoma, consistent with metastasis from the breast. The tumor was composed of numerous irregular anastomosing vascular channels lined by atypical endothelial cells with enlarged nuclei (Fig. 2). The patient was administered weekly paclitaxel (wPTX) treatment in the same month.

### 2.2. Case 2

**Figure 3.**
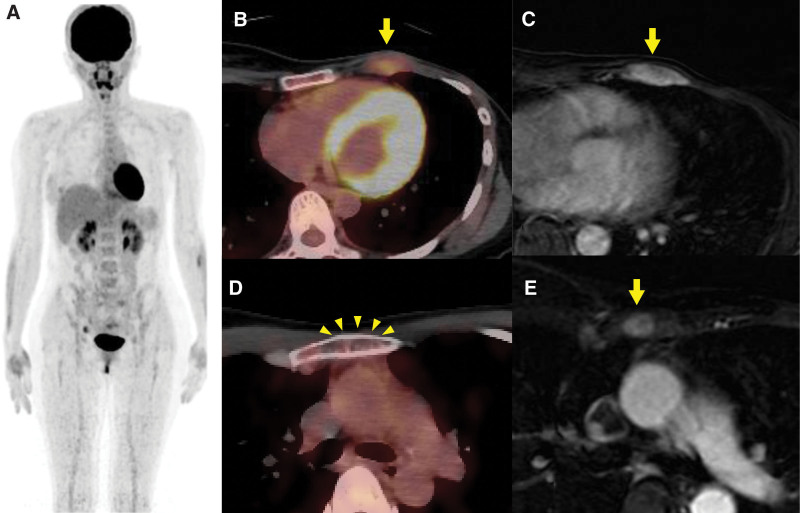
A 37-year-old woman was diagnosed with primary angiosarcoma of the breast and underwent mastectomy. During the postoperative follow-up, although the uptake was covered by the heart in the maximum intensity projection image (A), axial fluorine-18 fluorodeoxyglucose positron emission tomography/computed tomography (^18^F-FDG PET/CT) revealed abnormal uptake (SUVmax = 3.1) in the left third intercostal muscle (yellow arrow) (B). Gd-enhanced magnetic resonance imaging (MRI) also showed soft tissue mass in the left intercostal muscle (yellow arrow) in the axial image, suggesting local recurrence (C). Furthermore, axial Gd-enhanced MRI showed a 13-mm (diameter) contrast effect in the sternum (yellow arrow) (E), which suggested bone metastasis. Contrary to these findings, axial ^18^F-FDG PET/CT showed no uptake or osteolytic destruction in the sternum (D). SUV = standardized uptake value.

**Figure 4.**
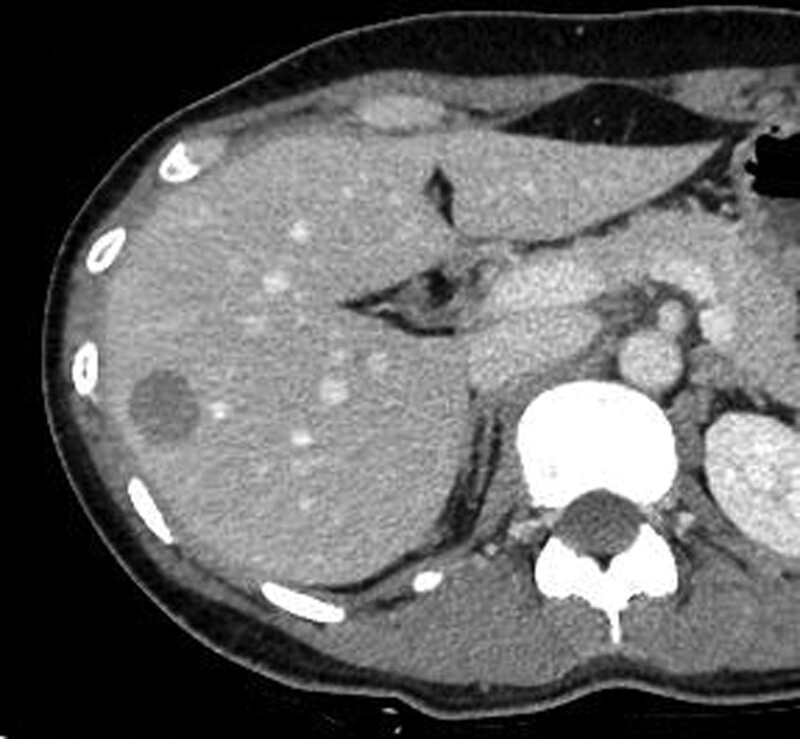
Axial enhanced computed tomography (CT) suggested a 20-mm contrast-impaired area in S5/6 of the liver (yellow arrow). Liver biopsy revealed that this lesion was consistent with angiosarcoma metastasis.

**Figure 5.**
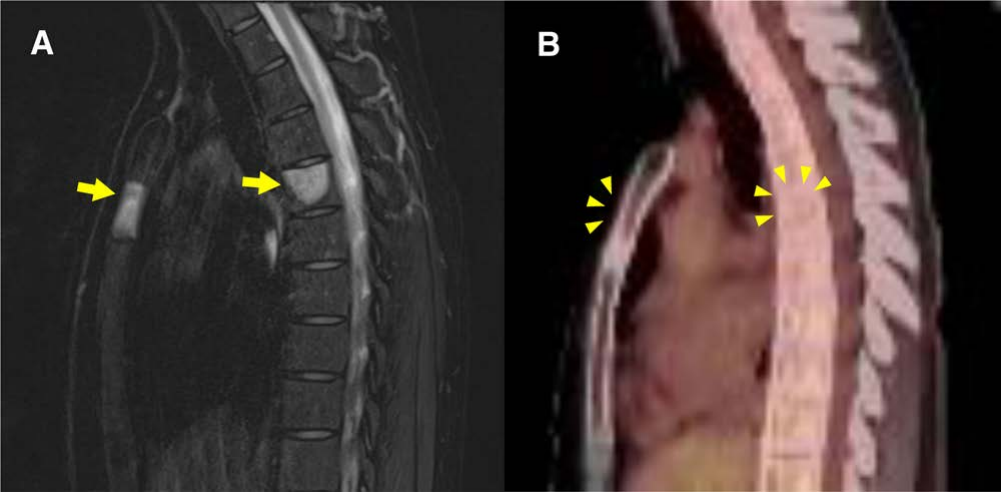
Sagittal Gd-enhanced magnetic resonance imaging (MRI) showed prolonged contrast effects in the sternum and the fifth thoracic vertebra (yellow arrows), suggesting multiple bone metastases (A). However, sagittal fluorine-18 fluorodeoxyglucose positron emission tomography/computed tomography (^18^F-FDG PET/CT) found no uptake or osteolytic destruction (yellow arrowheads), which is inconsistent with the MRI findings (B).

A 37-year-old woman presented with gradually enlarging mass in her left breast for the past 4 years. The patient had never been diagnosed with diabetes mellitus. One and a half years later, the patient was diagnosed with primary angiosarcoma of the breast, without any distant metastases, and underwent a mastectomy. Two and a half years after the mastectomy, during postoperative follow-up, a local recurrence in the left intercostal muscle was detected on ^18^F-FDG PET/CT (Fig. 3A and B). MRI indicated local recurrence (Fig.3C) in the left intercostal muscle, and additionally indicated a bone metastasis in the sternum (Fig. 3E), which showed no uptake on ^18^F-FDG PET/CT (Fig. 3D). Furthermore, enhanced CT detected a liver tumor (Fig. 4). Liver biopsy revealed that the liver tumor was consistent with angiosarcoma metastasis. Finally, the patient was diagnosed with local recurrence of the left intercostal muscle and distant metastases to the sternum and the liver, resulting from primary angiosarcoma of the left breast. wPTX was initiated as a salvage chemotherapy. After the second course of wPTX, MRI showed multiple bone metastases (the sternum and the fifth thoracic vertebra) (Fig. 5A). However, ^18^F-FDG PET/CT indicated no obvious uptake in the multiple bone metastatic lesions (Fig. 5B).

## 3. Discussion

^18^F-FDG PET/CT is considered useful not only for localization but also for the differentiation of vascular tumors, such as angiosarcoma, hemangioma, and epithelioid hemangioendothelioma, according to differences in FDG uptake patterns. It has been reported that standardized uptake value (SUV) max can be used to evaluate the status of vascular tumors.^[15]^ Several reports have demonstrated that ^18^F-FDG PET/CT could be used for pathological and prognostic evaluation of angiosarcoma, as well as its localization. Kato et al^[7]^ reported that the SUVmax at initial presentation correlated with the grading and overall survival of angiosarcoma. Umemura et al^[16]^ reported that cutaneous angiosarcomas with a higher SUVmax at the initial diagnosis had a significantly poorer prognosis than those with a lower SUVmax. Recently, Chen et al^[17]^ demonstrated that ^18^F-FDG PET/CT was highly sensitive and specific for detecting bone metastases of angiosarcoma, especially occult bone metastases, and that bone metastases detected by ^18^F-FDG PET/CT were poor prognostic factors for angiosarcoma. Considering its impact on the treatment plan as well as the patients’ quality of life or activities of daily living, such as pain and impending fracture, accurate early diagnosis of bone metastasis of angiosarcoma is important.

Generally, bone metastases on ^18^F-FDG PET/CT are detected by abnormal FDG uptake accompanied by osteolytic destruction, which is often consistent with MRI findings.^[17]^ In the 2 cases presented here, however, MRI showed multiple bone lesions, while ^18^F-FDG PET/CT detected neither uptake nor osteolytic destruction. To the best of our knowledge, there has been only 1 report of false-negative findings of bone metastases of angiosarcoma on ^18^F-FDG PET/CT. Among the 19 cases of angiosarcoma analyzed by Chen et al, bone metastases were found in 10 cases; however, 2 of them showed no uptake on ^18^F-FDG PET/CT, nor did they show osteolytic destruction. Chen et al^[17]^ did not mention the primary site of angiosarcoma in the 2 cases presenting false-negative findings. In both cases presented herein, the primary sites were angiosarcomas of the breast, which suggests that the primary site of angiosarcoma is related to false-negative results of bone metastases on ^18^F-FDG PET/CT.

Regarding the false-negative findings of bone metastases of sarcomas on ^18^F-FDG PET/CT, Aryal et al^[18]^ compared the accuracy of ^18^F-FDG PET/CT, bone scintigraphy, and whole-body MRI for staging diagnosis in 54 patients with osteosarcoma and Ewing sarcoma and found no significant difference among the 3 methods. On the other hand, Bosma et al^[19]^ examined the ability of MRI and ^18^F-FDG PET/CT to detect bone metastases in 112 bone specimens obtained from 20 patients with Ewing sarcoma. They found that ^18^F-FDG PET/CT tended to show false-negative findings of bone metastases compared to MRI when bone marrow hematopoiesis was active. This may be because the boundary between bone metastases and normal bone is unclear. The authors also noted that the variables of “active bone marrow hematopoiesis,” “under chemotherapy,” and “small tumor size” significantly reduced the detection rate of bone metastases on ^18^F-FDG PET/CT compared to MRI.

In both cases detailed in this case series, relatively small metastatic bone lesions with preserved bone trabecular structure may be collated with false-negative results on ^18^F-FDG PET/CT. Especially in Case 2, the ^18^F-FDG PET/CTs were performed during chemotherapy, which is consistent with one of the conditions for false-negative findings, as reported by Bosma et al.^[19]^

Although ^18^F-FDG PET/CT is frequently used in the evaluation of distant metastases and clinical staging of angiosarcomas, the possibility of false-negative findings should be considered, especially in the evaluation of bone metastases. Follow-up imaging studies, including MRI or open biopsy, should be performed.

## Acknowledgments

We would like to thank Hiroko Miyata for management of our department and organizing data.

## Author contributions

**Conceptualization:** Akane Ariga, Seiichi Matsumoto.

**Data curation:** Akane Ariga, Seiichi Matsumoto.

**Formal analysis:** Akane Ariga, Seiichi Matsumoto.

**Funding acquisition:** Keisuke Ae.

**Investigation:** Akane Ariga, Kyoko Yamashita, Takashi Terauchi, Keisuke Ae.

**Project administration:** Keisuke Ae.

**Resources:** Taisuke Tanizawa, Keiko Hayakawa, Yusuke Minami, Masanori Saito, Norio Kurosawa, Kyoko Yamashita, Takashi Terauchi.

**Supervision:** Seiichi Matsumoto.

**Visualization:** Akane Ariga.

**Writing – original draft:** Akane Ariga.

**Writing – review & editing:** Akane Ariga.

## References

[1] Young RJ Brown NJ Reed MW. Angiosarcoma. Lancet Oncol. 2010;11:983–91. 20537949 10.1016/S1470-2045(10)70023-1

[2] Antonescu C. Malignant vascular tumors – an update. Mod Pathol. 2014;27(Suppl 1):S30–8. 24384851 10.1038/modpathol.2013.176

[3] Lahat G Dhuka AR Hallevi H. Angiosarcoma: clinical and molecular insights. Ann Surg. 2010;251:1098–106. 20485141 10.1097/SLA.0b013e3181dbb75a

[4] Cassidy RJ Switchenko JM Yushak ML. The importance of surgery in scalp angiosarcomas. Surg Oncol. 2018;27:A3–8. 30237037 10.1016/j.suronc.2018.07.010 PMC6261443

[5] Buehler D Rice SR Moody JS. Angiosarcoma outcomes and prognostic factors: a 25-year single institution experience. Am J Clin Oncol. 2014;37:473–9. 23428947 10.1097/COC.0b013e31827e4e7b PMC3664266

[6] Fayette J Martin E Piperno-Neumann S. Angiosarcomas, a heterogeneous group of sarcomas with specific behavior depending on primary site: a retrospective study of 161 cases. Ann Oncol. 2007;18:2030–6. 17974557 10.1093/annonc/mdm381

[7] Kato A Nakamoto Y Ishimori T. Prognostic value of quantitative parameters of ^18^F-FDG PET/CT for patients with angiosarcoma. AJR Am J Roentgenol. 2020;214:649–57. 31939696 10.2214/AJR.19.21635

[8] Ren S Wang Y Wang Z. Survival predictors of metastatic angiosarcomas: a surveillance, epidemiology, and end results program population-based retrospective study. BMC Cancer. 2020;20:778. 32811474 10.1186/s12885-020-07300-7 PMC7437028

[9] Hori Y Funabashi N Miyauchi H. Angiosarcoma in the right atria demonstrated by fusion images of multislice computed tomography and positron emission tomography using F-18 fluoro-deoxyglucose. Int J Cardiol. 2007;123:e15–7. 17316847 10.1016/j.ijcard.2006.11.093

[10] Freudenberg LS Rosenbaum SJ Schulte-Herbrüggen J. Diagnosis of a cardiac angiosarcoma by fluorine-18 fluorodeoxyglucose positron emission tomography. Eur Radiol. 2002;12(Suppl 3):S158–61. 12522630 10.1007/s00330-002-1478-z

[11] Oe A Habu D Kawabe J. A case of diffuse hepatic angiosarcoma diagnosed by FDG-PET. Ann Nucl Med. 2005;19:519–21. 16248391 10.1007/BF02985582

[12] Lin E. Diagnosis of venous angiosarcoma by FDG PET/CT. Clin Nucl Med. 2008;33:66–7. 18097267 10.1097/RLU.0b013e318148b217

[13] Tokmak E Ozkan E Yağci S. F18-FDG PET/CT scanning in angiosarcoma: report of two cases. Mol Imag Radionucl Ther. 2011;20:63–6. 10.4274/MIRT.020397 PMC3590947 23486298

[14] Vasanawala MS Wang Y Quon A. F-18 fluorodeoxyglucose PET/CT as an imaging tool for staging and restaging cutaneous angiosarcoma of the scalp. Clin Nucl Med. 2006;31:534–7. 16921276 10.1097/01.rlu.0000233073.12599.0a

[15] Lee WW So Y Kang SY. F-18 fluorodeoxyglucose positron emission tomography for differential diagnosis and prognosis prediction of vascular tumors. Oncol Lett. 2017;14:665–72. 28693219 10.3892/ol.2017.6192 PMC5494675

[16] Umemura H Yamasaki O Kaji T. Prognostic value of ^18^F-fluorodeoxyglucose positron emission tomography/computed tomography in patients with cutaneous angiosarcoma: a retrospective study of 18 cases. J Dermatol. 2017;44:1046–9. 28370268 10.1111/1346-8138.13839

[17] Chen D Tang M Lv S. Prognostic usefulness of clinical features and pretreatment ^18^F-FDG PET/CT metabolic parameters in patients with angiosarcoma. Quant Imaging Med Surg. 2022;12:2792–804. 35502366 10.21037/qims-21-563 PMC9014154

[18] Aryal A Kumar VS Shamim SA. What is the comparative ability of 18F-FDG PET/CT, 99mTc-MDP skeletal scintigraphy, and whole-body MRI as a staging investigation to detect skeletal metastases in patients with osteosarcoma and Ewing sarcoma? Clin Orthop Relat Res. 2021;479:1768–79. 33635285 10.1097/CORR.0000000000001681 PMC8277296

[19] Bosma SE Vriens D Gelderblom H. F-FDG PET-CT versus MRI for detection of skeletal metastasis in Ewing sarcoma. Skeletal Radiol. 2019;48:1735–46. 31016339 10.1007/s00256-019-03192-2 PMC6776481

